# Impact of Comorbidity on Fatality Rate of Patients with Middle East Respiratory Syndrome

**DOI:** 10.1038/s41598-017-10402-1

**Published:** 2017-09-12

**Authors:** Ya-Min Yang, Chen-Yang Hsu, Chao-Chih Lai, Ming-Fang Yen, Paul S. Wikramaratna, Hsiu-Hsi Chen, Tsung-Hsi Wang

**Affiliations:** 1 Division of Biostatistics, College of Public Health, National Taiwan University, Taipei, Taiwan; 2 Emergency Department, Taipei City Hospital, Ren-Ai Branch, Taipei, Taiwan; 3 School of Oral Hygiene, College of Oral Medicine, Taipei Medical University, Taipei, Taiwan; 4 Institute of Evolutionary Biology, University of Edinburgh, Edinburgh, United Kingdom; 5 Ministry of Health and Welfare, Taipei, Taiwan; 6 School of Medicine, National Yangming University, Taipei, Taiwan

## Abstract

To date, 1841 cases of Middle East respiratory syndrome coronavirus (MERS-CoV) infection have been reported worldwide, with 652 deaths. We used a publically available case line list to explore the effect of relevant factors, notably underlying comorbidities, on fatal outcome of Middle East respiratory syndrome (MERS) cases up to the end of October 2016. A Bayesian Weibull proportional hazards regression model was used to assess the effect of comorbidity, age, epidemic period and sex on the fatality rate of MERS cases and its variation across countries. The crude fatality rate of MERS cases was 32.1% (95% credibility interval (CI): 29.9%, 34.3%). Notably, the incremental change of daily death rate was most prominent during the first week since disease onset with an average increase of 13%, but then stabilized in the remaining two weeks when it only increased 3% on average. Neither sex, nor country of infection were found to have a significant impact on fatality rates after taking into account the age and comorbidity status of patients. After adjusting for age, epidemic period, MERS patients with comorbidity had around 4 times the risk for fatal infection than those without (adjusted hazard ratio of 3.74 (95% CI: 2.57, 5.67)).

## Introduction

A Middle East respiratory syndrome (MERS) case was first reported in 2012 in Saudi Arabia where the novel virus Middle East respiratory syndrome coronavirus (MERS-CoV) was identified^1, 2^. Although an animal reservoir has been proposed as the ultimate source of infection^3^, evidence of interhuman transmissibility in community clusters^3–6^, among hospital contacts^7, 8^, and among health care workers^8^ have raised concern about the pandemic risk of this emerging infectious disease^9^. These fears were underlined by an outbreak of MERS that took place in South Korea in May 2015 where the index case was a 68-year-old male with a travel history to four countries in the Middle East^10, 11^. By the time this outbreak was declared over on 28th July in 2015, it had claimed the lives of 36 of the 186 confirmed cases^11^.

According to previous studies, the emerging disease has only modest transmissibility^9, 12^. A genomic study has also revealed the genetic diversity in case clusters, suggesting sporadic virus introduction from an animal reservoir^13^. This epidemiological evidence indicates that MERS-CoV probably has low pandemic potential, although adaptation towards improved human-to-human transmission remains a concern^9, 14^.

Case-fatality rates also remain troubling. For example, an outbreak South Korean outbreak demonstrated a high case-fatality rate in the first epidemic wave^10, 14, 15^; analysis using outbreak data of a district hospital in South Korea till Jun, 2015 showed a median incubation period of 6 days (95% confidence interval (CI): 4–7) and a fatality rate of 16%^16^. The outbreak of MERS in South Korea and China in 2015 only underlined the importance of disease surveillance and disease control strategies, especially in hospitals^10^. This is reminiscent of the 2003 outbreak of severe acute respiratory syndrome (SARS)^15, 17^, also caused by a coronavirus, with a case fatality rate around 10%, which was even higher at 46% in cases with comorbidities^15^.

Case fatality rates between 20% and 70% have been reported by other studies^12, 15, 16, 18–20^ with age, sex, and comorbid condition seemingly important cofactors^12, 15, 16, 19, 21, 22^. However, lack of data on comorbidities has hampered efforts to systematically consider its clinical importance; for example a recent study by Lessler *et al*. was able to assess the impact of age on outcome amongst Saudi patients, but their dataset did not contain information on whether patients had underlying comorbidities^22^. Furthermore, the case fatality rate may also vary from country to country due to the difference in disease surveillance system and health care system^12, 21^.

Here, we use data from a publically available case line list of global MERS cases where the reported comorbidity status of patients has been recorded, to try to assess the impact of comorbidity on fatality rate, while simultaneously adjusting for age and sex in a Bayesian multilevel survival model. We also assess the variation in case-fatality rate across countries.

## Results

A total of 1743 MERS cases that had been recorded in the line list at the end of October, 2016, including 1393 from KSA, 80 from UAE, 186 from South Korea, and 84 from other 11 countries, were enrolled for analysis. The majority of patients in other countries had travelled to KSA and UAE prior to the onset MERS. Descriptive results on demographic characteristics, comorbidity, and contact patterns are provided in Table 1. Fatal cases in patients with MERS were older (*P* < 0.001) and predominated in males (*P* < 0.001). Among the 1223 cases where comorbid conditions were reported, patients with comorbidity were at elevated risk for being dead compared with those without (11.1% vs 48.3%, *P* < 0.001). Note that cases with unknown comorbid conditions were similar to those without. When the history of contact patterns were categorized into camel contact, other animals, and presumed human, there was no statistical difference in outcome across these contact patterns (*P* = 0.476). The fatality rate was higher in the period before Mar. 20, 2014, when compared to after (*P* = 0.004). The fatality rate was the highest in KSA (34.7%), followed by others (32.1%), South Korea (19.9%), and the lowest in UAE (15.0%), however, the difference in fatality rates across countries was statistically significant (*P* < 0.001).

**Table 1:** Characteristics of reported MERS Cases^a^.

Variable		Death		Alive		Total	*P*
Age (Mean (SD))^b^		61.08	(17.05)	46.94	(17.00)	—	<0.001
Sex^b^	Female	147	(25.43)	431	(74.57)	578	<0.001
	Male	410	(35.71)	738	(64.29)	1148	
Comorbidity	No	31	(11.11)	248	(88.89)	279	<0.001
	Yes	456	(48.31)	488	(51.69)	944	
	NR	72	(13.85)	448	(86.15)	520	
Contact pattern	Camel	58	(34.94)	108	(65.06)	166	0.476
	Other animal	10	(40.00)	15	(60.00)	25	
	Human	491	(31.64)	1061	(68.36)	1552	
Country	KSA	483	(34.67)	910	(65.33)	1393	<0.001
	UAE	12	(15.00)	68	(85.00)	80	
	South Korea	37	(19.89)	149	(80.11)	186	
	Others^c^	27	(32.14)	57	(67.86)	84	
Epidemic period^d^	Initial	92	(40.35)	136	(59.65)	228	0.004
	Later	467	(30.83)	1048	(69.17)	1515	
Total		559	(32.07)	1184	(67.93)	1743	

^a^Data are frequency (percentage) unless otherwise stated, NR: not reported, KSA: Kingdom of Saudi Arabia, UAE: United Arab Emirates. ^b^9 (1 death) and 17 cases (2 death) with missing information on age and sex, respectively. ^c^Including France (1 case, 0 death), Iran (8 cases, 2 death), Italy (2 cases, 0 death), Jordan (35 cases, 10 death), Kuwait (4 cases, 2 death), Lebanon (1 case, 0 death), Oman (11 cases, 5 death), Qatar (17 cases, 6 death), Tunisia (2 cases, 0 death), United Kingdom (2 cases, 1 death), Yemen (1 case, 0 death). ^d^Epidemic period was classified as initial (before 2014/03/20) and later (after 2014/03/21) period.

**Figure 1:**
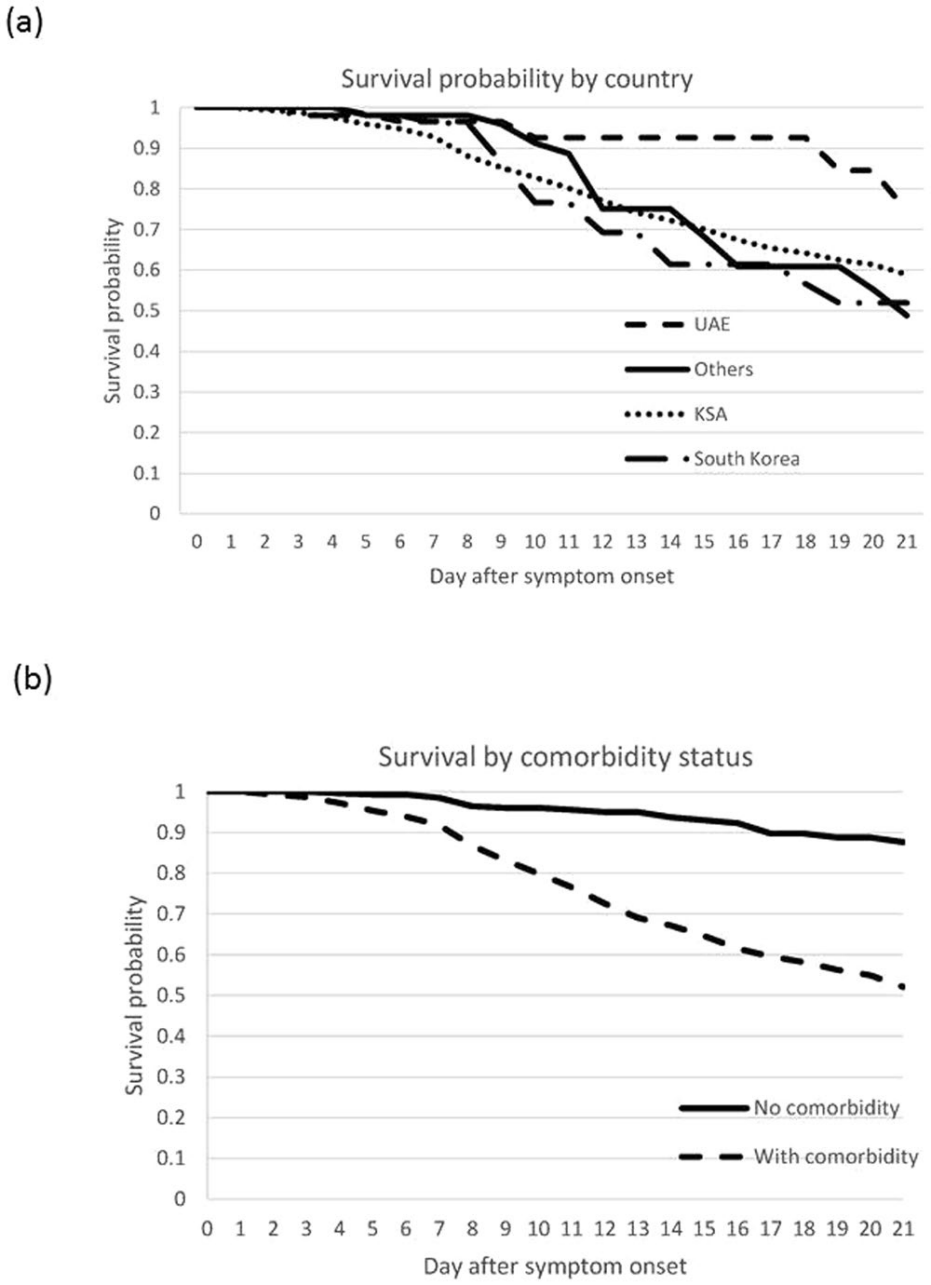
Survival Probability of MERS Cases by Countries (**a**) and Comorbidity Status (**b**).

The results of univariate analysis are listed in the first column of Table 2. The crude survival curves by countries and comorbidity status are presented in panel (a) and (b) in Fig. 1, respectively. The survival curves for KSA, other countries, and South Korea were similar with each other, with the survival probabilities lower than UAE (Fig. 1(a)), corresponding to the higher HRs of these countries listed in the results of univariate analysis in the first column of Table 2. The survival probability of subjects without comorbidity at 21 days was around 90%, as opposed to 50% for those with comorbidities (Fig. 1(b)).

**Table 2:** Risk of Death Among MERS Cases by Characteristics of Subjects^a^.

Variable		Univariate analysis			Multiple variable analysis, Fixed effect model					
		*HR/Estimate*	*95% CI*		*aHR/Estimate* ^b^	*95% CI*		*aHR/Estimate* ^c^	*95% CI*	
Intercept		—	—		−6.82	(−7.42	−6.22)	−6.87	(−7.74,	−6.31)
Age		1.03	(1.02,	1.04)	1.02	(1.01,	1.03)	1.02	(1.01,	1.03)
Sex	*Male/Female*	1.25	(1.02,	1.55)	—	—		1.10	(0.90,	1.36)
Comorbidity	5.39	(3.77,	7.90)	3.74	(2.57,	5.67)	3.70	(2.52,	5.60)	
Epidemic later period ^d^		0.74	(0.58,	0.96)	0.68	(0.54,	0.88)	0.68	(0.53,	0.88)
Contact pattern	*Other animal* Reference				—	—		—	—	
	*Camel*	0.73	(0.39,	1.48)	—	—		—	—	
	*Human*	0.97	(0.54,	1.88)	—	—		—	—	
Country	*South Korea*	1.91	(0.97,	3.91)	—	—		—	—	
	*KSA*	1.56	(1.01,	2.67)	—	—		—	—	
	*UAE*	0.73	(0.31,	1.64)	—	—		—	—	
	*Others*	Reference			—	—		—	—	

Abbreviations: aHR, adjusted hazard ratio; CI, credibility interval; HR, hazard ratio. ^a^1216 subjects (470 deaths) with information on age and comorbidity were included in the analysis. ^b^Model: T~Weibull (λ,*v*), *h*(*t*) = λ*v*t^(*v*−1)^, λ = exp{*α* + *β*_*0*_ × age + *β*_*1*_ × comorbidity + *β*_2_ × epidemic period}, shape parameter *v* = 1.38 (95% CI: 1.29–1.47) ^c^Model: T~Weibull (λ,*v*), *h*(*t*) = λ*v*t^(*v*−1)^, λ = exp{*α* + *β*_*0*_ × age + *β*_*1*_ × comorbidity + *β*_*2*_ × epidemic period + *β*_3_ × sex}, shape parameter *v* = 1.38 (95% CI: 1.29–1.47). ^d^Epidemic period was classified as initial (before 2014/03/20) and later (after 2014/03/21) period.

**Figure 2:**
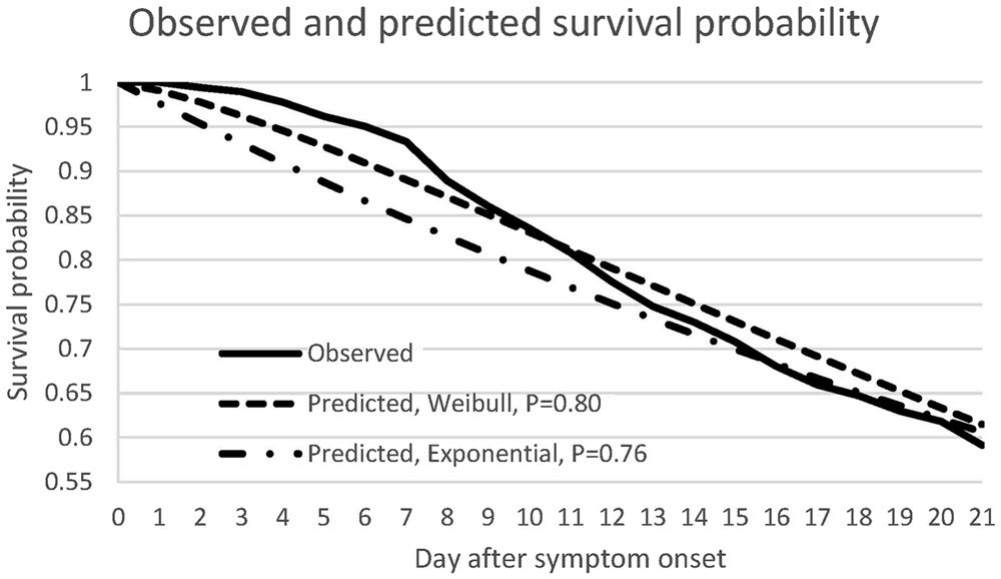
Observed and Predicted Survival Probability Curve Based on Weibull and Exponential Distribution.

Although both models provided acceptable fits to the data (likelihood ratio test; *P* = 0.80 for the Weibull distribution and *P* = 0.76 for the exponential distribution, Fig. 2), model selection by DIC values (see Supplementary material G) suggests that the model based on the Weibull distribution was superior. The Weibull distribution was thus selected as the shape of the baseline mortality of the MERS cases for the evaluation of the fatality rate.

**Figure 3:**
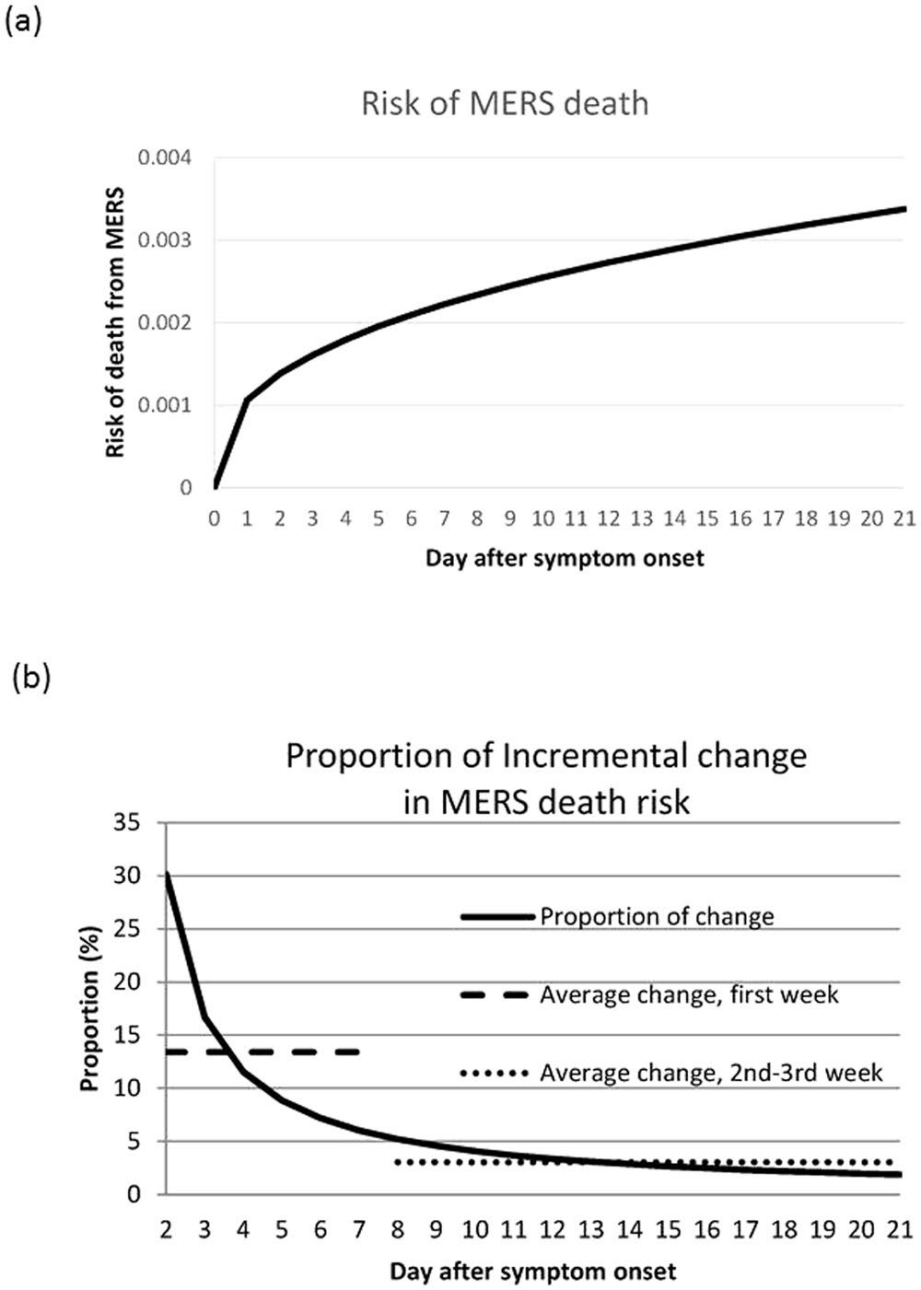
Daily Death Risk (**a**) and Incremental Change of Death Risk (**b**) of MERS Cases.

The estimated results of the multivariate analysis using Weibull proportional hazards regression model analysis with fixed effect are listed in Table 2. The shape parameter was greater than 1 (Shape (*v*): 1.38, 95% CI: 1.29, 1.47) indicating the daily risk of dying from MERS increased with time at a decreasing rate (Fig. 3(a)). Notably, the incremental change of daily death rate was most prominent during the first week since disease onset with an average of 13% and then stabilized for the remaining two weeks with an average of 3% (Fig. 3(b) and Supplementary material H). Although male patients had a higher crude risk of dying from MERS, the effect of sex was not significant after taking into account the effect of comorbidity and age in the fixed effect model (adjusted HR (aHR): 1.10; 95% CI: 0.90, 1.36) and for all models (by the comparison of DIC values, Supplementary G). After adjusting for age and epidemic period (initial two years), the effect of comorbidity on fatality rate remained statistically significant (aHR: 3.74; 95% CI: 2.57, 5.67). The incremental increase in age conferred an increased risk of MERS death with the aHR estimated as 1.02 (95% CI: 1.01, 1.03). In addition, the risk of death was decreased in the later epidemic period (aHR: 0.68; 95% CI: 0.54, 0.88). The effect of age, epidemic period and comorbidity on MERS death was found to be consistent when using an alternative logistic model (see Supplementary material I.1).

We also assessed the variation of baseline MERS fatality rate and the effect of comorbidity on MERS fatality among countries based on a multilevel Weibull proportion hazards regression model. By the comparison of DIC values (Supplementary material G), the baseline variation in MERS fatality was not significant after considering the effect of age, epidemic period and comorbidity in the model. There was also no apparent heterogeneity in the effect of comorbidity across countries. The estimated results taking into account the effect of heterogeneity across countries on baseline fatality rate (random intercept model) and that of the effect of comorbidity (random slope model) were provided in Supplementary material I.2.

**Figure 4:**
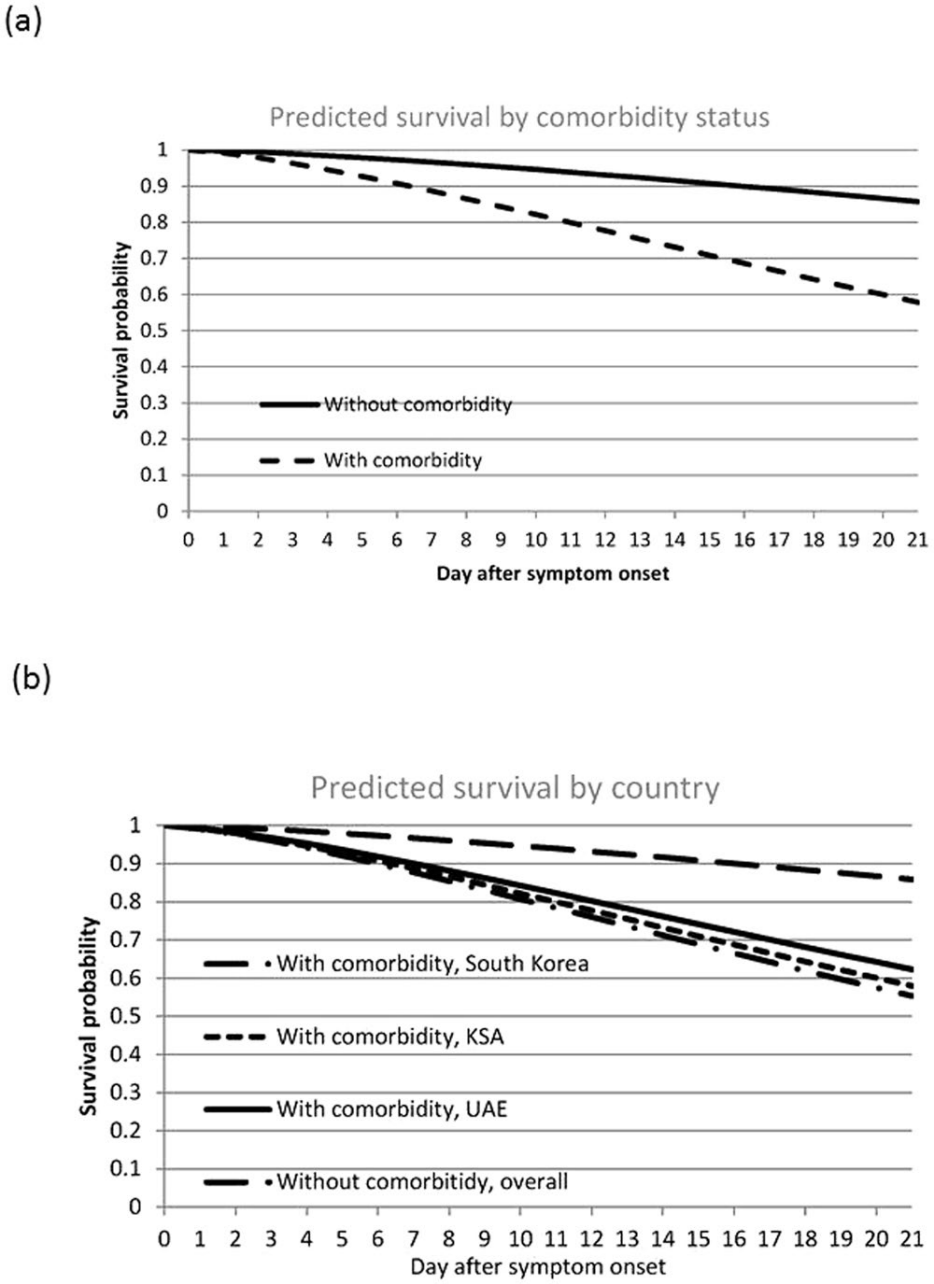
Predicted Survival Probability for Subjects With and Without Comorbidity Adjusting for Age Based on Fixed Effect (**a**) and Random Slope Model (**b**) for South Korea, KSA, and UAE.

The predicted survivals based on the results of Weibull regression model with fixed effect and random slope were presented in Fig. 4(a) and (b), respectively. The overall survival for MERS cases without comorbidity at 21st day was 86.6% while that for cases with comorbidity was 60.0% based on the predictions from the fixed effect model for age and comorbidity.

## Discussion

The fatality rate of MERS cases based on the data till the end of October, 2016 was 32.1%. We quantified the effect of comorbidity on fatality rate of MERS cases by using a multilevel Weibull proportional hazards regression model. Those with comorbid conditions and/or who were older had a significantly higher fatality rate, but the difference in fatality rate among countries of disease onset was not significant. We also found that there was a pronounced difference in fatality rates between what we described as the early and later period of the epidemic. The risk of MERS death soared during the first week since disease onset with an average of 13% daily increment. The average daily increase in the risk of MERS death reduced to 3% for the remaining two weeks.

This pattern of decreasing risk of death as time from onset increases was also reported during the epidemic of SARS^23, 24^. Applying survival analysis enabled us to detect the change in the force of fatal outcome by time reflected by the shape of baseline hazard showing a trend of increasing by decreasing rate and an improved fit by applying Bayesian proportional hazards regression model with Weibull distribution to observed data (Fig. 3 and Supplementary material H). More importantly, our approach demonstrated the framework of predicting the prognosis of MERS cases with the consideration of individual characteristics, country-level heterogeneities, and time-varying change to the risk of MERS death.

**Figure 5:**
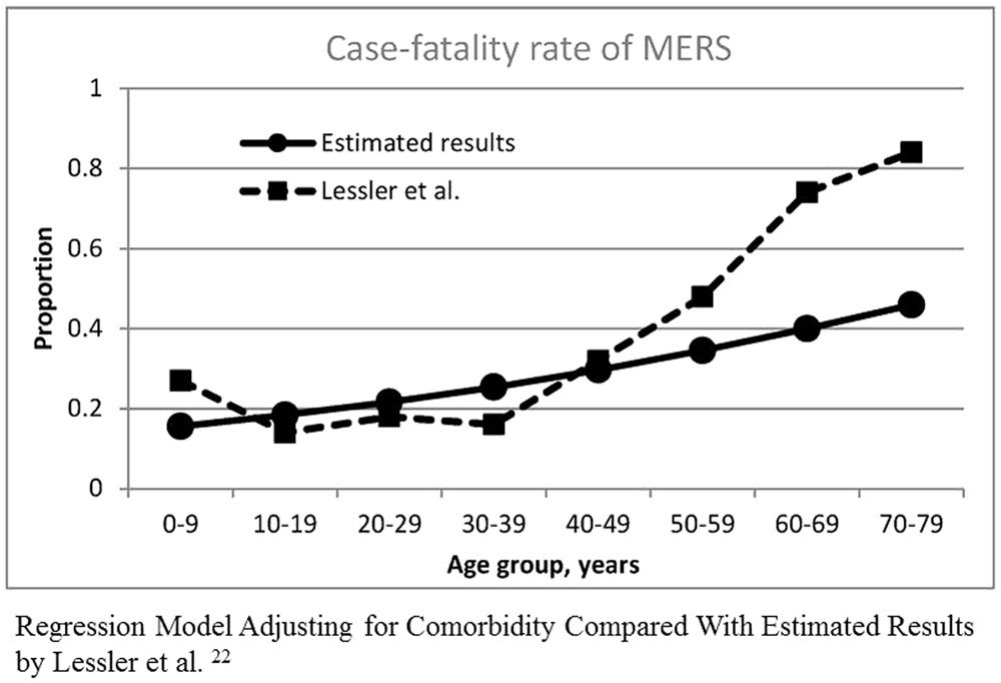
Case Fatality Rate by Age Groups Based on Weibull Proportional Hazards.

The case fatality rates by age groups were plotted using the estimated results based on the Weibull proportion hazards regression model with fixed effect adjusting for comorbidity and was compared with the results of Lessler *et al*.^22^ in Fig. 5. Our estimated results were consistent with that reported by Lessler *et al*. till the age group of 40–49 years, after which our case fatality rates appear markedly lower (35% vs 48% for 50–59 year olds, 40% vs 74% for 60–69 year olds, and 46% vs 84% for those over 70 years old)^22^. This discrepancy almost certainly arises because we have been able to take account of patient’s comorbidity status, and these older patients are more likely to have comorbidities. Our data also includes MERS cases from outside KSA, but given the lack of evidence for variation in the impact of comorbidities across countries, this does not seem plausible as a major reason for these observed differences.

We found that the baseline fatality rate and also the variation on the effect of comorbidity on MERS death between countries was not significant after taking the effect of age, comorbidity and epidemic period into account (Fig. 4(b) and Supplementary material G). In the absence of change in disease characteristics induced by mutation for MERS-CoV^9, 14^ the main factors that may have major effect on case fatality after adjusting for underlying disease status (comorbidity), and age including the quality of medical treatment and time of initiation of medical intervention^12, 25, 26^. The non-significant variation of fatality rate across countries we found here after taking into account the characteristic of MERS patients suggests that any variation in disease monitoring, contact tracing, case identification and thus timing of medical intervention across countries has had minimal clinical impact on average. However, the increasing survival rate we noted in the later epidemic period in our study could indicate that the quality and/or timing of medical treatment has generally improved as the epidemic has progressed. The potential causes of this require further study, but could be due to increased awareness, especially enhanced reporting of mild and asymptomatic cases. Conversely, it seems unlikely that this change is associated with a single mutation in the virus because several different MERS CoV phylogenetic clades have circulated in this period^15^. We also note that the case fatality rate of Zaire Ebola virus has similarly decreased with time^27^.

We also found no significant differences in outcome based on sex once we had accounted for both age and underlying comorbidities. This may indicate that the higher fatality rates for males detected in the univariate analysis, could be because males are more likely to have these underlying comorbidities, or because elderly males with underlying comorbidities are more likely to exposed to infection than equivalent females.

The source of infection appeared to have no impact of MERS fatality rates in our study, a conclusion further supported by the observation that the patterns of transmission have varied amongst countries^28^ (e.g. almost purely nosocomial in South Korea^29^) while the fatality rate has not. Besides, in the absence of evidence of the gene mutation of MERS-CoV^9, 14^, there ought not to be different clinical manifestations for subjects who contract the disease either by contact with camel, some other animal or humans.

Possession of comorbidities increased the mortality rate of MERS patients in this study. This is in line with previous studies of other diseases: for example, influenza patients with chronic cardiovascular disease^30^, or SARS patients with diabetes mellitus, end-stage renal disease^15^. The relationship between comorbidities and emerging infectious diseases or gene association has been studied in SARS^31–33^, and found that some comorbidities are strongly associated with SARS, such as immunological, neurological, metabolic and dermatologic disease^31^. The mechanisms underlying such mortality enhancing interactions are not always clear, but an already weakened host may have fewer options available to counteract the new infection^34^.

Rivers *et al*. have previously reported similar findings which MERS cases with increasing age and underlying comorbidity were at higher risk for death and severe disease by using an earlier snapshot of this data^20^. Neverthelss, the work here differs in four respects. First, we have used a Weibull proportional hazards rather than Poisson regression, which has allowed us to investigate the incremental change in daily death rate over time. Second, we have not chosen to exclude the outbreak in South Korea as they did on the grounds that it was “unique.” Rather we have tried to explicitly model its potentially unique nature by allowing for variation in fatality rates across countries, finding no evidence that the characteristics of the South Korean outbreak being different from that seen anywhere else. Third, we have more than a year’s extra data to analyse. Finally, these factors have combined to lead to our conclusion that those with a later time of infection onset had lower risk in our multivariate analysis, but this only showed in the univariate analysis of Rivers’s study.

Despite these differences, both studies point to comorbidities as having a substantial impact on MERS prognosis. Unfortunately, we have been unable to perform a more detailed analysis: all we know is that having an underlying health problem significantly elevates the risk of death. This is because although our analysis including age, sex, and comorbidity as the major factors associated with fatal outcome, other relevant factors such as biochemical values, type of comorbidities and treatments were not included because such information is not typically publically reported. However, the failure to detect an improvement in the fit of our models by DIC when we applied random intercept and random slope to the impact of comorbidities across countries (Supplementary material G) suggests a homogenous definition for comorbid conditions among countries. Furthermore, the proposed method provides greater insight into the course of MERS cases^35^ and also the prediction of prognosis based on the time-varying risk on fatality.

In conclusion, the existence of comorbid condition in MERS cases predicts a fourfold risk of fatal outcome compared with those without, alongside a generally elevated increase in fatality rate with age. Although sex and country of disease onset appear important in univariate analysis of outcome, these factors disappear once age and the presence or absence of comorbidities is taken into account. Finally, we offer evidence that the fatality rate for MERS has been 0.68-fold lower in the period since March 2014 than before.

## Methods

### Data sources

Data on reported cases of MERS cases (Epidemic curve was showed in supplementary material A), from March 1, 2012 to Oct. 31, 2016, were derived from a web-based line list maintained by Rambaut and Wikramaratna^36, 37^ with reference to the published literatures on the cases reports^1, 38–40^ and cases series of outbreak^4–7, 21^, communications^41, 42^, and the announcement of the disease information system of each country and WHO^11^. Based on the collected data, Rambaut and Wikramaratna^37^ provided an up-to-date analysis on spatial, temporal and epidemiological information with interactive web-based tool and the line list of MERS cases were followed and updated till the end of October 2016 including cases of the outbreak in South Korea^37^. The updated data in A copy of the relevant parts of the case list used here is available in Supplementary material as a separate file (CaseList.docx).

### Study design

The collected data consisted of a disease cohort of MERS cases with follow up till the occurrence of fatal outcome or discharge, thus a prospective cohort design. In some cases, information on infection outcome is unavailable (censored) either because the case is too recent or because no publically available update was reported. For reported cases with missing onset date, the date of symptom onset was calculated by subtraction the reported date by the median of the time period between onset date and reported date at each country^12^.

### Variable definition

In addition to report date and final outcome, information on age, sex, comorbidity, animal contact pattern, and dates of disease onset, hospitalization, discharge, and death of MERS cases were also included in the line list of the data file^36^. Contact with animals was recorded as either specifically with camels, or more generally with animals, as reported. For the purposes of our analysis here a human source was otherwise assumed. Cases with reported underlying medical conditions such as diabetes mellitus, cardiovascular disease, renal disease, and pulmonary disease were defined as having comorbidity and those reported as previously healthy were defined as having no comorbidity. For cases without available information on previous medical status, the comorbid condition was then categorized as not reported (NR)^36^. In addition to individual characteristics, information on the country of disease onset including KSA (Kingdom of Saudi Arabia), UAE (United Arab Emirates), South Korea and other countries including France, Iran, Italy, Jordan, Kuwait, Lebanon, Oman, Qatar, Tunisia, United Kingdom, and Yemen were also collected.

### Statistical analysis

Descriptive analysis on characteristics of reported MERS cases including age, sex, contact pattern and country of disease onset for fatal and survival cases were tabulated. The comparison of continuous variable such as age between mortality and survival cases was assessed by *t*-test and those for categorical data were evaluated by chi-squared test. The comparisons of the means of the variables (age, sex, comorbidity, contact pattern, and country) among those who were alive and death were based on a two-tailed test with a significance level of 0.05.

The effects of individual characteristics including age, sex, comorbidity, and contact pattern on the risk of fatality rate of MERS cases were evaluated by using Bayesian proportional hazards regression model^43^ (Supplementary material G). Since the fatality rate of MERS could vary from country to country, we also used a random intercept model to test for this. We also used a random slope model to assess possible variation on the effect of comorbidity on fatality rate across countries.

The full conditional posterior distribution was derived by using directed acyclic graphic (DAG) model using WinBUGS environment. In addition, the non-informative prior distribution of *N*(0, 10^4^) for the regression coefficients, non-informative prior distribution of *gamma* (0.01, 0.01) for the shape parameter (*v*) of Weibull distribution and the inverse of variance parameters of random intercept and random slope (*σ* ^2^_*β*_). The evaluation of parameters was based on 15000 samples with the thinning interval of 3 after a burn-in period of 5000, which gives 5000 posterior samples. The analysis was carried out using WinBUGS^44^.

The baseline hazard function, *h*_0_(*t*), was chosen between exponential and Weibull distribution according to the Deviance information criterion (DIC) of each model; the same is true for deciding whether to include random effect parameters. The smaller the DIC value is, the better the model performance.

**Figure 6:**
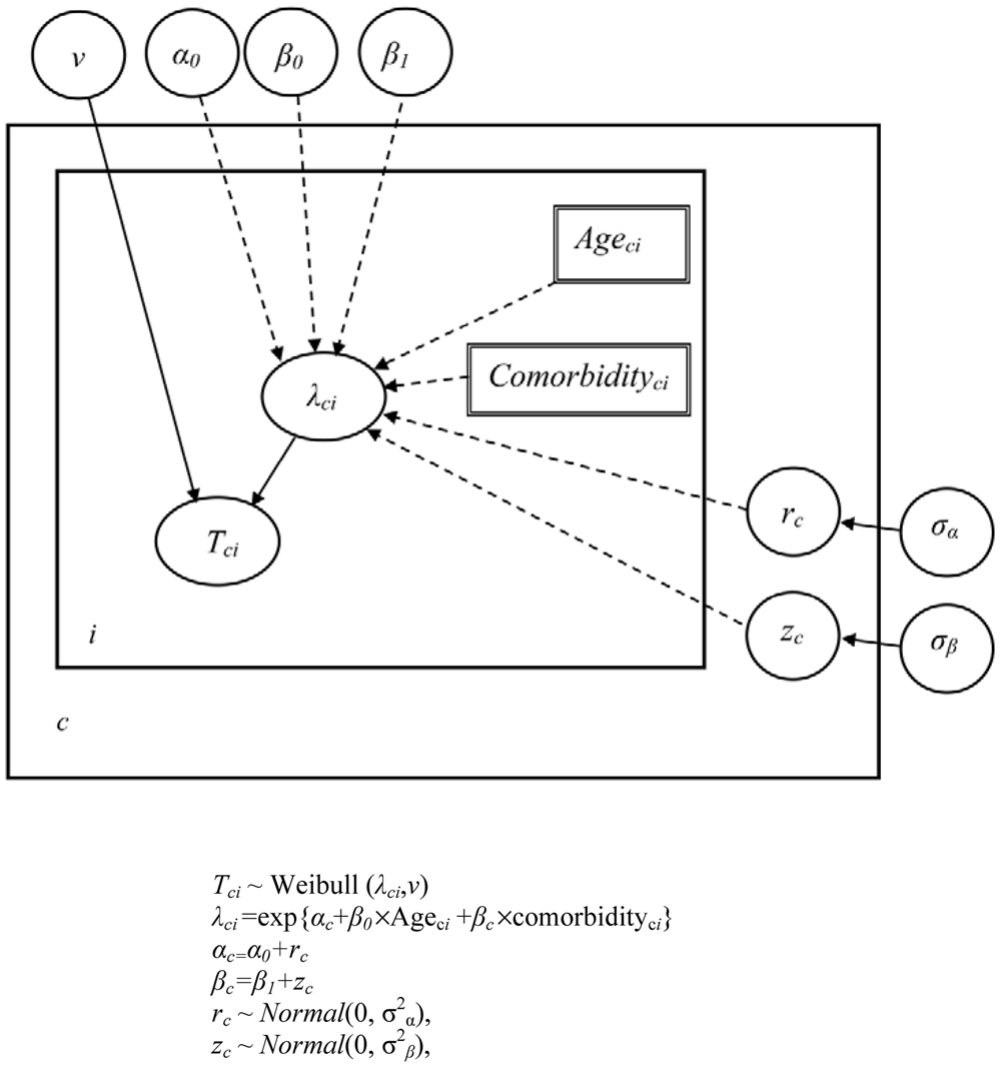
Directed Acyclic Graphic Model of Weibull Proportional Hazards Regression Model with Random Intercept and Random Slope.

A DAG model depicting the proportional hazards regression model using Weibull distributions as the baseline hazard function incorporating the heterogeneities on both the baseline hazard of MERS death and the effect of comorbidity is presented in Fig. 6.

Further details on model specification of the proportional hazards regression model with random effects are given in Supplementary material B. An example WinBUGS code of Weibull proportional hazards regression model with random intercept and random slope is given in Supplementary material C. Parameter convergence was assessed based on the trace plots and the Gelman-Rubin statistics of three chains^45^. An illustration of trace plots, autocorrelation plots, and Gelman-Rubin statistics for Weibull proportional hazards regression model with random intercept and random slope was provided in Supplementary materials D, E, and F, respectively.

## Electronic supplementary material

Supplementary Material

Dataset 1
