# Supplementary material for: Impact of Comorbidity on Fatality Rate of Patients with Middle East Respiratory Syndrome
Source: Sci Rep. 2017 Sep 12;7:11307. doi: 10.1038/s41598-017-10402-1 (PMC5596001; doi:10.1038/s41598-017-10402-1)
Supplement: Supplementary file 1 — Supplementary Material [file 41598_2017_10402_MOESM1_ESM.doc]

**Impact of Comorbidity on Fatality Rate of Patients with Middle East Respiratory Syndrome**

Ya-Min Yang1, Chen-Yang Hsu1, Chao-Chih Lai2**†**, Ming-Fang Yen3, Paul S. Wikramaratna4, Hsiu-Hsi Chen1 and Tsung-Hsi Wang5*

1Division of Biostatistics, College of Public Health, National Taiwan University, Taipei, Taiwan

2Emergency Department, Taipei City Hospital, Ren-Ai Branch, Taipei,Taiwan 3School of Oral Hygiene, College of Oral Medicine, Taipei Medical University, Taipei, Taiwan

4Institute of Evolutionary Biology, University of Edinburgh, Edinburgh, United Kingdom

5Ministry of Health and Welfare, Taiwan (Tsung-Hsi Wang); Department of Public Health & Medical Humanities, Institute of Public Health, National Yangming University, Taipei, Taiwan

*Reprint Request and Correspondence to Tsung-Hsi Wang, Ministry of Health and Welfare, Taipei, Taiwan. No.488, Sec. 6, Zhongxiao E. Rd., Nangang Dist., Taipei City 115, Taiwan (email: joyce12wang@gmail.com)

**†**Corresponding Author During Submission: Chao-Chih Lai, Emergency department, Taipei City Hospital, Ren-Ai branch, Taipei, Taiwan. No. 10, Sec. 4, Ren-Ai Road, Taipei, Taiwan. (Chaochin@ms1.hinet.net)

**Supplementary Material**

**A. Epidemic curve of MERS in South Korea and the Middle East**

**(a) Epidemic curve of MERS in South Korea**

**
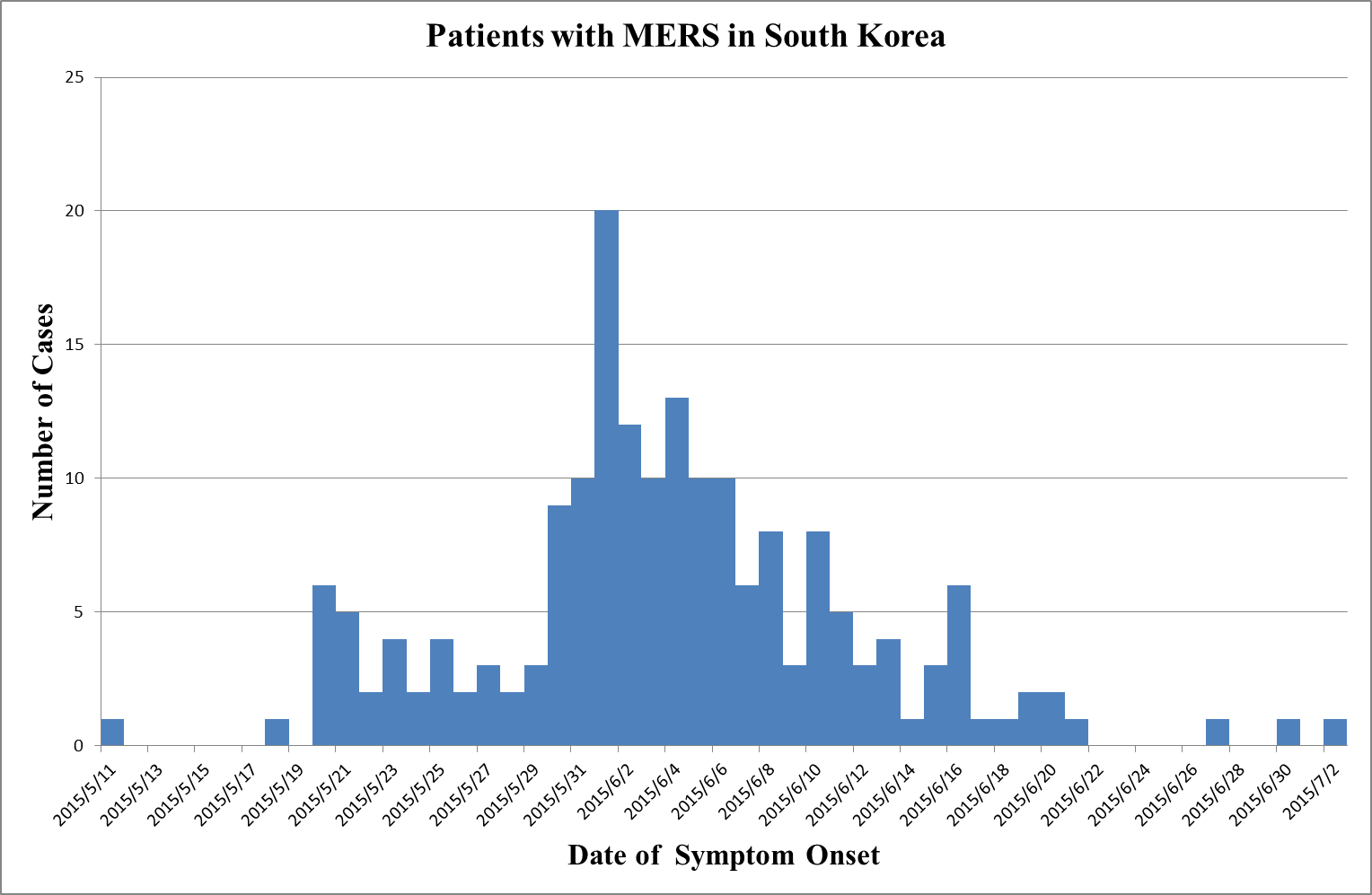
**

**(b) Epidemic curve of MERS in KSA, UAE and others**

**(**KSA: Kingdom of Saudi Arabia, UAE: United Arab Emirates, Others: France, Iran, Italy, Jordan, Kuwait, Lebanon, Oman, Qatar, Tunisia, United Kingdom, Yemen**)**

**
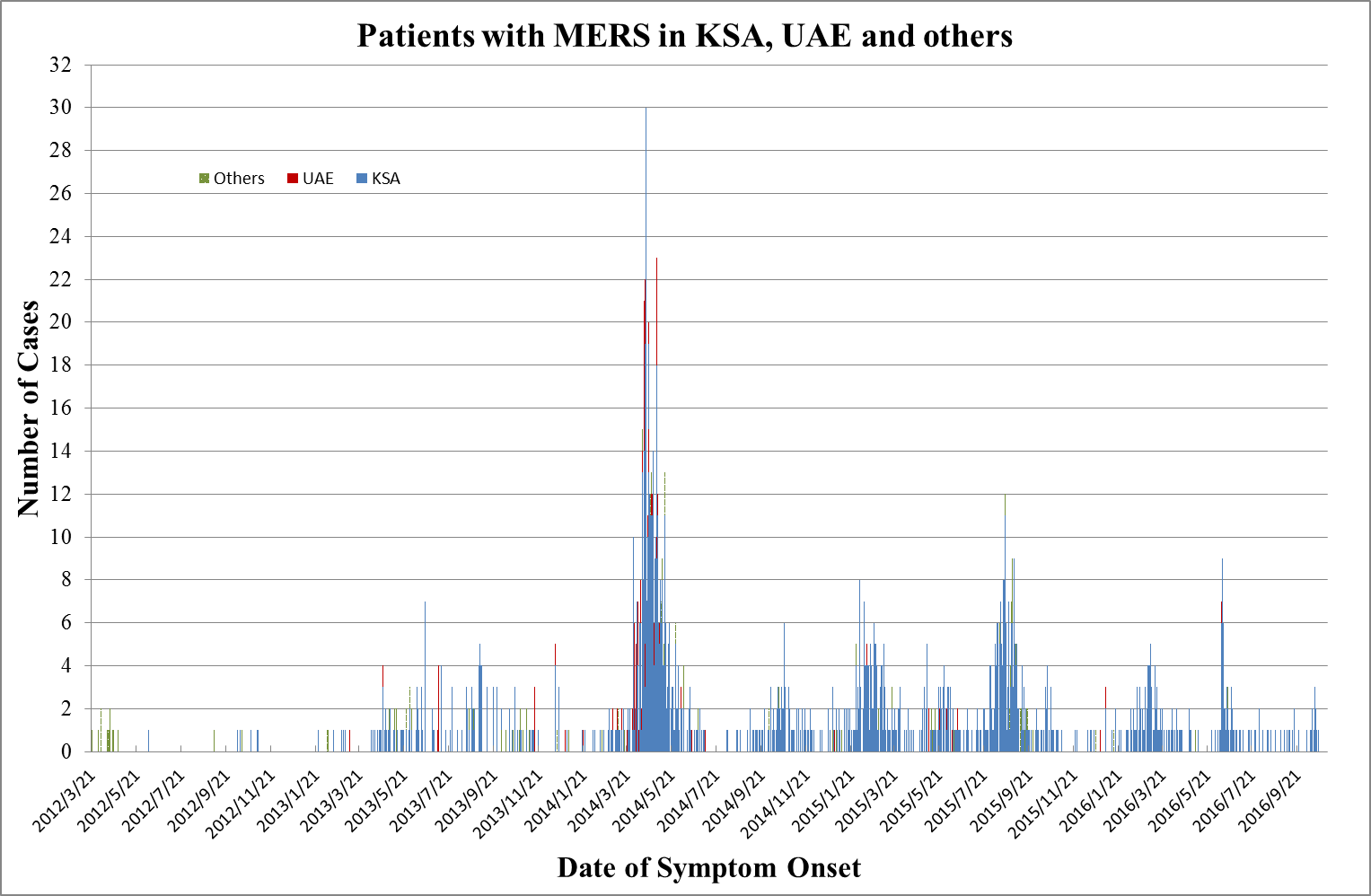
**

Data on reported cases of MERS cases (Epidemic curve was showed in supplementary material A), from March 1, 2012 to Oct. 31, 2016. In order to see the impact of different epidemic periods, we classified the whole epidemic period into early and late periods. The symptom onset of first case was on Mar. 21, 2012. Therefore, we defined early epidemic period as initial two years, from Mar. 21, 2012 to Mar. 20, 2014. In the early epidemic period, there were sporadic cases or a minor epidemic of MERS.

**B. Model Specification of Bayesian Proportion Hazards Regression Model With Random Effect.**

The effect of relevant factors were incorporated by using the product of baseline hazard and the exponent of linear combination of these factors written as *h*(*t*)=*h*0(*t*)exp(**βX**), where **X** denotes the vector of covariates including age, sex, comorbidity, and contact pattern and **β** is the vector of corresponding effect size in terms of regression coefficients. The more detrimental effect brought by an effect, the larger the regression coefficient and thus the risk of death from MERS, *h*(*t*), will be. Considering the baseline hazard of MERS death, *h*0(*t*), two parametric forms, exponential and Weibull distribution were assessed corresponding to the clinical scenarios of constant and time-varying risk of MERS death, respectively. Since the fatality rate of MERS could vary from country to country, we also used a random intercept model to test for this. We also used a random slope model to assess possible variation on the effect of comorbidity on fatality rate across countries.

A proportional hazards regression model can be specified as follows

$h(t)=h_{0}(t)\exp(\mathbf{\beta X})$, (1)

where **X** is an array of covariates including age and comorbidity and **β** is the array of regression coefficient corresponding to the effect of the covariates. The baseline hazard, *h0*(*t*), can be specified according to the distribution of survival time (*T*). For exponential distribution, a constant hazard is used and equation (1) can be written as λ=exp(α+**βX**). The likelihood contribution from a subject died from MERS occurred at time *t* and that survive longer than time *t* (censored observation) can be derived as follows

$\Pr(T=t)=\lambda\exp(-\lambda t)$, (2)

and

$\Pr(T\geq t)=\exp(-\lambda t)$, (3)

respectively. Considering a survival time with Weibull distribution, the equation (2) and (3) can be extended as follows

$\Pr(T=t)=\lambda\gamma t^{\gamma-1}\exp(-\lambda t^{\gamma})$, (4)

and

$\Pr(T\geq t)=\exp(-\lambda t^{\gamma})$ (5)

respectively.

Equation (1) can be extended based on the Bayesian framework to incorporate random intercept and also random slope. Denote the observed time in the empirical data for subject *i* of country *c* by *Tci­*. The Weibull proportion hazards regression model with random intercept and random slope was specified as follows

*Tci* ~ Weibull (*λci*,*v*)

*λci* =exp{*αc*+*β1*$\times$Agec*i* +*βc*$\times$comorbidityc*i* +*βe*$\times$epidemic periode*i* }

*αc=α0+rc*

*βc=**β0+zc*

*rc­* ~ *Normal*(0, σ2α­),

*zc­* ~ *Normal*(0, σ2*β*­), (6)

where *αc* and *βc* is the parameter of random intercept and random slope, respectively. The common intercept *α0* represents the average baseline hazard of the occurrence of death and the normal distributed random variable *rc*with a zero mean and the variance σ2α­ captures the heterogeneity at country level. The common effect of comorbidity on the risk of death was represented by *β0*. The normal distributed random variable *βc* with zero mean and variance σ2*β*­ was allowing for changing from country to country to capture the heterogeneity in the effect of comorbidity between countries.

For subjects with events (death) observed, the contribution of such an observation was in the form of probability density function (PDF) specified by (2) and (4). For those who survived during the periods of observation, the recovery date or 2015-Jun-15, whichever came first, was used as the time of end-of-observation. Such an observation were censored and the contribution to likelihood function was in the form of survival function (Pr (T$\geq$*t*)) as specified by (3) and (5). Although we did not have the information on the exact time of dying from MERS for those who survived during the period of observation, such kind of data provided information on the probability of surviving longer than the time observed (Pr(T$\geq$*t*)).

The non-informative prior distribution of *N*(0, 104) was used for the regression coefficients of age, sex, common intercept and the common effect of comorbidity. Non-informative prior distribution of *gamma* (0.01, 0.01) was specified for the shape parameter (*v*) of Weibull distribution and the inverse of variance parameters of random intercept (*σ*2*α*) and random slope (*σ*2*β*). The full conditional posterior distribution was derived by using directed acyclic graphic (DAG) model using WinBUGS environment. The evaluation of parameters was based on 15000 samples with the thinning interval of 3 after a burn-in period of 5000, which gives 5000 posterior samples. The analysis was carried out using WinBUGS 1.

The estimated results of shape v, in Table 2 represented the Weibull shape parameter of the baseline MERS death risk. This is due to the results of comparing the DIC values (Supplementary material G) between the model using exponential and Weibull distribution as the baseline risk function, h0(t), specified in the model h(t)=h0(t)exp(βX). In contrast to exponential distribution, Weibull distribution embrace the benefit of depicting time-varying risk of MERS death by including the shape parameter in the baseline risk function. The shape parameter considering the risk of MERS death was estimated as 1.38 (95% CI: 1.29-1.47) which indicates a significant deviation from the constant death risk assumption adopted in the exponential distribution. An increased MERS death risk with time at a decreasing rate was revealed from the estimated result. This interpretation was provided in Results section.

**C. WinBUGS Code of Applying Bayesian Proportional Hazards Regression Model With Random Effect Using Weibull Distribution.**

model

{ for(i in 1 : 13) {

for(j in 1 : id[i]) {

stime[i, j] ~ dweib(alpha, lamda[i,j])I(tcent[i, j],)

lamda[i,j]<-exp(ba*age[i,j]+bcs[i]*comorbidity1[i,j]+bp*period[I,j]+r[i]);

}

bcs[i]~dnorm(bcomo, taus)

r[i]~dnorm(b0, tau)

}

b0~dnorm(0.0, 1.0E-4);

ba~dnorm(0.0, 1.0E-4);

bcomo~dnorm(0.0, 1.0E-4);

bp~dnorm(0.0, 1.0E-4);

alpha ~ dgamma(0.01,0.01)

taus ~ dgamma(0.01,0.01);

tau ~ dgamma(0.01,0.01);

sigmas0 <- sqrt(1/taus);

sigma0 <- sqrt(1/tau);

}

**D. Trace Plots of Parameters Applying Bayesian Proportional Hazards Regression Model With Random Effect Using Weibull Distribution With Three Chains.**

Weibull shape (*v*)

Common intercept (*α0*)

Age (*β0*)

Comorbidity, common effect (*β1*)

Epidemic period, (*β2*)

Variation in intercept (σα­)

Variation in the effect of comorbidity (σ*β*­)

**E. Autocorrelation Plot of Parameters Applying Bayesian Proportional Hazards Regression Model With Random Effect Using Weibull Distribution.**

Weibull shape (*v*) Common intercept (*α0*)

Age (*β0*) Comorbidity, common effect (*β1*)

Epidemic period, (β2)

Variation in intercept (σα­) Variation in the effect of comorbidity (σ*β*­)

**F. Gelman-Rubin Convergence Criterion of Parameters Applying Bayesian Proportional Hazards Regression Model With Random Effect Using Weibull Distribution.**

Weibull shape (*v*) Common intercept (*α0*)

Age (*β0*) Comorbidity, common effect (*β1*)

Epidemic period, (β2)

Variation in intercept (σα­) Variation in the effect of comorbidity (σ*β*­)

**G. Deviance Information Criterion (DIC) Value of Models.**

| **Baseline hazard** | **Model** | **Covariate** | **DIC** |
| --- | --- | --- | --- |
| **Exponential** | Fixed effect only | No covariates | 4453.28 |
|  |  | Age, Comorbidity | 4313.33 |
|  |  | Age, Comorbidity, Sex | 4313.83 |
|  | Random intercept | Age, Comorbidity | 4315.22 |
|  |  | Age, Comorbidity, Sex | 4315.53 |
|  | Random slope | Age, Comorbidity | 4314.45 |
|  |  | Age, Comorbidity, Sex | 4314.98 |
|  | Random intercept, random slope | Age, Comorbidity | 4315.85 |
|  |  | Age, Comorbidity, Sex | 4316.17 |
| **Weibull** | Fixed effect only | No covariates | 4399.29 |
|  |  | Age, Comorbidity | 4238.79 |
|  |  | Age, Comorbidity, Sex | 4239.80 |
|  |  | Age, Comorbidity, Epidemic Period | 4232.38 |
|  |  | Age, Comorbidity, Sex, Epidemic Period | 4233.32 |
|  | Random intercept | Age, Comorbidity | 4240.33 |
|  |  | Age, Comorbidity, Sex | 4241.53 |
|  |  | Age, Comorbidity, Epidemic Period | 4232.45 |
|  |  | Age, Comorbidity, Sex, Epidemic Period | 4233.08 |
|  | Random slope | Age, Comorbidity | 4239.61 |
|  |  | Age, Comorbidity, Sex | 4240.35 |
|  |  | Age, Comorbidity, Epidemic Period | 4232.71 |
|  |  | Age, Comorbidity, Sex, Epidemic Period | 4270.61 |
|  | Random intercept, random slope | Age, Comorbidity | 4239.93 |
|  |  | Age, Comorbidity, Sex | 4241.01 |
|  |  | Age, Comorbidity, Epidemic Period | 4230.40 |
|  |  | Age, Comorbidity, Sex, Epidemic Period | 4231.41 |

We fist assessed the effect of age, sex, epidemic period and comorbidity on MERS death risk. Considering the model including age, sex, epidemic period and comorbidity as covariate, the model with fixed effect of age, comorbidity, and epidemic period using Weibull distribution as baseline MERS death risk was the one with smallest DIC value (4232.38), followed by the random slope model (4232.71) and random intercept model (4232.45) with a non-significant difference. The random intercept or random slop model was not statistical significant. Although the random slope with random intercept model further brought down the DIC value, the difference was not significant. However, the fixed model including age, epidemic period and comorbidity was the best parsimonious model because sex was not a significant factor. Therefore, the result provides no evidence in favor of variation in survival probability across countries given the status of comorbidity and age of the MERS patient.

**H. Daily Risk and Incremental Change of MERS Death Risk Based on Estimated Results of Multivariate Weibull Proportional Hazards Regression Model.**

| Day since MERS onset | Risk (hazard) of MERS death (‰) | Incremental change | Proportion of change (%) | Average (%) |
| --- | --- | --- | --- | --- |
| 0 | 0 |  | - | 13.4 |
| 1 | 1.127 | 1.127 | - |
| 2 | 1.467 | 0.340 | 30.1 |
| 3 | 1.711 | 0.244 | 16.7 |
| 4 | 1.909 | 0.198 | 11.6 |
| 5 | 2.078 | 0.169 | 8.8 |
| 6 | 2.227 | 0.149 | 7.2 |
| 7 | 2.361 | 0.134 | 6.0 |
| 8 | 2.484 | 0.123 | 5.2 | 3.0 |
| 9 | 2.598 | 0.114 | 4.6 |
| 10 | 2.704 | 0.106 | 4.1 |
| 11 | 2.804 | 0.100 | 3.7 |
| 12 | 2.898 | 0.094 | 3.4 |
| 13 | 2.988 | 0.090 | 3.1 |
| 14 | 3.073 | 0.085 | 2.9 |
| 15 | 3.155 | 0.082 | 2.7 |
| 16 | 3.233 | 0.078 | 2.5 |
| 17 | 3.308 | 0.075 | 2.3 |
| 18 | 3.381 | 0.073 | 2.2 |
| 19 | 3.451 | 0.070 | 2.1 |
| 20 | 3.519 | 0.068 | 2.0 |
| 21 | 3.585 | 0.066 | 1.9 |

**I.1 Estimated Results Applying Binomial Model And Weibull Proportional Hazards Regression Model.**

The estimated results applying the binomial model with fixed effect are listed in the following Table. For the convenience of comparison, the estimated results applying the Weibull proportional hazards regression model are also listed (second column). The adjusted odds ratios (aOR) of age and comorbidity by using the binomial model were consistent with that derived by using Weibull proportional hazards regression model.

|  | Multiple variables analysis, Fixed effect model | | | | | |
| --- | --- | --- | --- | --- | --- | --- |
|  | **Binomial model** | | | **Weibull model** | | |
|  | *aOR/estimate* | *95% CI* | | *aHR/estimate* | *95% CI* | |
| **Intercept** | -2.64 | -3.26 | -2.05 | -6.82 | -7.42, | -6.22 |
| **Age** | 1.028 | 1.019, | 1.036 | 1.019 | 1.013, | 1.025 |
| **Comorbidity** | 3.83 | 2.51, | 5.99 | 3.74 | 2.57, | 5.67 |
| **Later Epidemic Period** | 0.58 | 0.40 | 0.85 | 0.68 | 0.54 | 0.88 |
| **Shape** | - |  | - | 1.38 | 1.29, | 1.47 |

Epidemic period was classified as initial (before 2014/03/20) and later (after 2014/03/21) period.

Abbreviations: *a*HR, adjustedhazard ratio; CI, credible interval; HR, hazard ratio

**I.2 Estimated Results on Risk of Death Among MERS Cases Using Weibull Proportional Hazards Regression Model with Random Effect**a.

|  |  | **Univariate analysis** | | | **Multiple variables analysis** | | | | | | | |  |
| --- | --- | --- | --- | --- | --- | --- | --- | --- | --- | --- | --- | --- | --- |
|  |  |  |  |  | *Random intercept* b | | | | | *Random slope* c | | | |
|  |  | *HR/Estimate* | *95% CI* | | *aHR/Estimate* | | *95% CI* | | | *aHR/Estimate* | *95% CI* | | |
| **Intercept** |  | - | - |  | -6.81 | | -7.50, | | -6.15 | -6.78 | -7.40, | -6.25 | |
| **Age** |  | 1.030 | 1.024, | 1.035 | 1.019 | | 1.012, | | 1.024 | 1.018 | 1.013, | 1.025 | |
| **Sex** |  | 1.25 | 1.02, | 1.55 |  | | - | | - | - |  | - | |
| **Comorbidity** | | 5.39 | 3.77, | 7.90 | 3.57 | | 2.37, | | 5.44 | 3.85 | 2.35, | 6.46 | |
| **Epidemic Period** | | 0.74 | 0.58, | 0.96 | 0.65 | | 0.50 | | 0.85 | 0.67 | 0.52 | 0.87 | |
| **Random effect** | *Intercept* (σα)*-* | | - |  | 0.30 | | 0.08, | | 0.76 | - |  | - | |
|  | *Slope* (σβ) | - | - |  |  | | - | | - | 0.29 | 0.07, | 0.76 | |
| **Shape** | (*v*) | - | - |  | 1.38 | | 1.29, | | 1.48 | 1.38 | 1.29, | 1.47 | |
| **Contact pattern** | *Other animal Reference* | | | |  | | - | | - | - |  | - | |
|  | *Camel* | 0.73 | 0.39, | 1.48 |  | | - | | - | - |  | - | |
|  | *Human* | 0.97 | 0.54, | 1.88 |  | | - | | - | - |  | - | |
| **Country** | *South Korea* | 1.91 | 0.97, | 3.91 |  | - | | - | | - |  | - | |
|  | *KSA* | 1.56 | 1.01, | 2.67 |  | | - | | - | - |  | - | |
|  | *UAE* | 0.73 | 0.31, | 1.64 |  | | - | | - | - |  | - | |
|  | *Others* | *Reference* | | |  | | - | | - | - |  | - | |

Abbreviations: *a*HR, adjustedhazard ratio; CI, credible interval; HR, hazard ratio

a 1216 subjects (470 deaths) with information on age and comorbidity were included in the analysis.

b Model: T*ci*~Weibull (λ*c*,*v*), *hc*(*t*)= λ*cv*t(*v*-1), λ*c*=exp{(*α0*+ *rc* )+*β0*$\times$age*ci* +*β1*$\times$comorbidity*ci*+*β2*$\times$period*ci*},

*rc* ~ *Normal*(0, σ2α­), for country *c* and patient *i*

c Model: T*ci*~Weibull (λ*c*,*v*), *hc*(*t*)= λ*cv*t(*v*-1), λ*c*=exp{*α0*+*β0*$\times$age*ci* +(*β1+zc*)$\times$comorbidity*ci*+*β2*$\times$period*ci*},

*zc­* ~ *Normal*(0, σ2*β*­), for country *c* and patient *i*

**Reference**:

1 Lunn, D. J., Thomas, A., Best, N. & Spiegelhalter, D. WinBUGS - A Bayesian modelling framework: Concepts, structure, and extensibility*. Statistics and Computin***g** 10, 325-337 (2000).
